# Fungal pathogens—a sweet and sour treat for *toll*-like receptors

**DOI:** 10.3389/fcimb.2012.00142

**Published:** 2012-11-22

**Authors:** Christelle Bourgeois, Karl Kuchler

**Affiliations:** Medical University of Vienna, Max F. Perutz LaboratoriesVienna, Austria

**Keywords:** fungal pathogens, TLRs, phagocytes, APCs, hematopoietic cells, epithelial cells

## Abstract

Hundred-thousands of fungal species are present in our environment, including normal colonizers that constitute part of the human microbiota. The homeostasis of host-fungus interactions encompasses efficient fungal sensing, tolerance at mucosal surfaces, as well as antifungal defenses. Decrease in host immune fitness or increase in fungal burden may favor pathologies, ranging from superficial mucocutaneous diseases to invasive life-threatening fungal infections. Toll-like receptors (TLRs) are essential players in this balance, due to their ability to control both inflammatory and anti-inflammatory processes upon recognition of fungal-specific pathogen-associated molecular patterns (PAMPs). Certain members of the TLR family participate to the initial recognition of fungal PAMPs on the cell surface, as well as inside phagosomes of innate immune cells. Active signaling cascades in phagocytes ultimately enable fungus clearance and the release of cytokines that shape and instruct other innate immune cells and the adaptive immune system. Some TLRs cooperate with other pattern recognition receptors (PRRs) (e.g., C-type lectins and Galectins), thus allowing for a tailored immune response. The spatio-temporal and physiological contributions of individual TLRs in fungal infections remains ill-defined, although in humans, TLR gene polymorphisms have been linked to increased susceptibility to fungal infections. This review focuses entirely on the role of TLRs that control the host susceptibility to environmental fungi (e.g., *Aspergillus, Cryptoccocus*, and *Coccidoides*), as well as to the most frequent human fungal pathogens represented by the commensal *Candida* species. The emerging roles of TLRs in modulating host tolerance to fungi, and the strategies that evolved in some of these fungi to evade or use TLR recognition to their advantage will also be discussed, as well as their potential suitability as targets in vaccine therapies.

## Introduction

An estimated 1.5 million fungal species are present in the environment (Hube, 2009). Some of them have evolved as commensal colonizers of cutaneous and mucosal surfaces in humans. While only a few fungal microbes are actually true pathogens for healthy individuals, in Western societies opportunistic fungi can cause life-threatening infections in immunosuppressed individuals, ranging from superficial mucocutaneous disease to invasive deep-seated infections. In developing countries, fungal infections affect not only immunocompromised but also immunocompetent healthy individuals in region of endemic mycoses (Brown et al., 2012), with *Cryptococcus* species (spp.) representing the major human fungal pathogen (Del Poeta and Chaturvedi, 2012). The main fungal pathogens affecting humans comprise those ubiquitously present in the environmental fungi, *Aspergillus fumigatus*, *Cryptoccocus neoformans* and more recently *Cryptoccocus gatii*, *Histoplasma capsulatum*, *Coccidoides posadasii, Pneumocystis jirovecii* and the commensal *P. jiroveci* or the *Candida* spp. The rising incidence in fungal infections observed in the last decades correlates with increases in invasive medical interventions, long-term hospitalization and with large numbers of immunosuppressed patients due to acquired- (e.g., HIV infection) or treatment-induced immunodeficiency such as transplantation or anticancer therapy (Pfaller and Diekema, 2007, 2010). No obvious clinical symptoms distinguish invasive fungal infections from other microbial infections. Furthermore, clinical diagnoses pose a huge challenge, since current methods are not always reliable, speedy, accurate, or specific, in particular when speciation is required for efficient antifungal therapy. Thus, anti-fungal treatments are often delayed or inappropriately applied. Consequently, fungi stand out as the fourth main cause of hospital acquired infections in “at-risk” populations, despite availability of efficient but costly antifungal therapies (Perlin, 2011; Pfaller, 2012).

Several particularities distinguish fungal from viral or bacterial microbes in their interaction with host immune cells. For instance, many fungal pathogens are dimorphic and able to undergo morphogenesis upon environmental or host stimuli, which facilitates immune evasion or dissemination and niche occupancy in the host. Morphogenesis is hence considered a major virulence trait (Gow et al., 2012). Further, all fungal eukaryotes are protected by the cell wall, a highly complex and flexible meshwork of carbohydrate polymers such as mannans, β-glucans, and chitin interwoven in a protein matrix (Gow and Hube, 2012). Due to its physical properties and its plasticity, this unique structure confers strong protection against all kinds of environmental stresses, including immune cell attack. It also is a major source of fungal pathogen-associated molecular patterns (PAMPs) that mediate host-fungi interaction during recognition by immune cells (Levitz, 2010).

The major class of pattern recognition receptors (PRRs) known to be involved in sensing and recognition of fungal PAMPs comprise the C-type lectin receptor family recognizing glucan and mannan (such as Dectin-1, Dectin-2, Mincle, SIGNR, and mannose receptor), the scavenger receptors (such as CD5 and CD36), Galectin-3, and the Toll-like receptor (TLR) family (Romani, 2011). This review addresses TLRs recognizing fungal pathogens on hematopoietic and non-hematopoietic cells, recapitulating the most recent advances in the field. We shall reiterate the emerging concept of TLRs in shaping host-fungal relationships. Importantly, we will also discuss fundamental differences of TLR function in mouse and humans, since there is increasing evidence not only for cell-type specific responses, but also for species-specific distinct roles of TLRs in fungal immunity or tolerance.

## Fungal sensing by toll-like receptors

### Recognition of fungal PAMPs by surface and phagosomal TLRs

**Figure 1:**
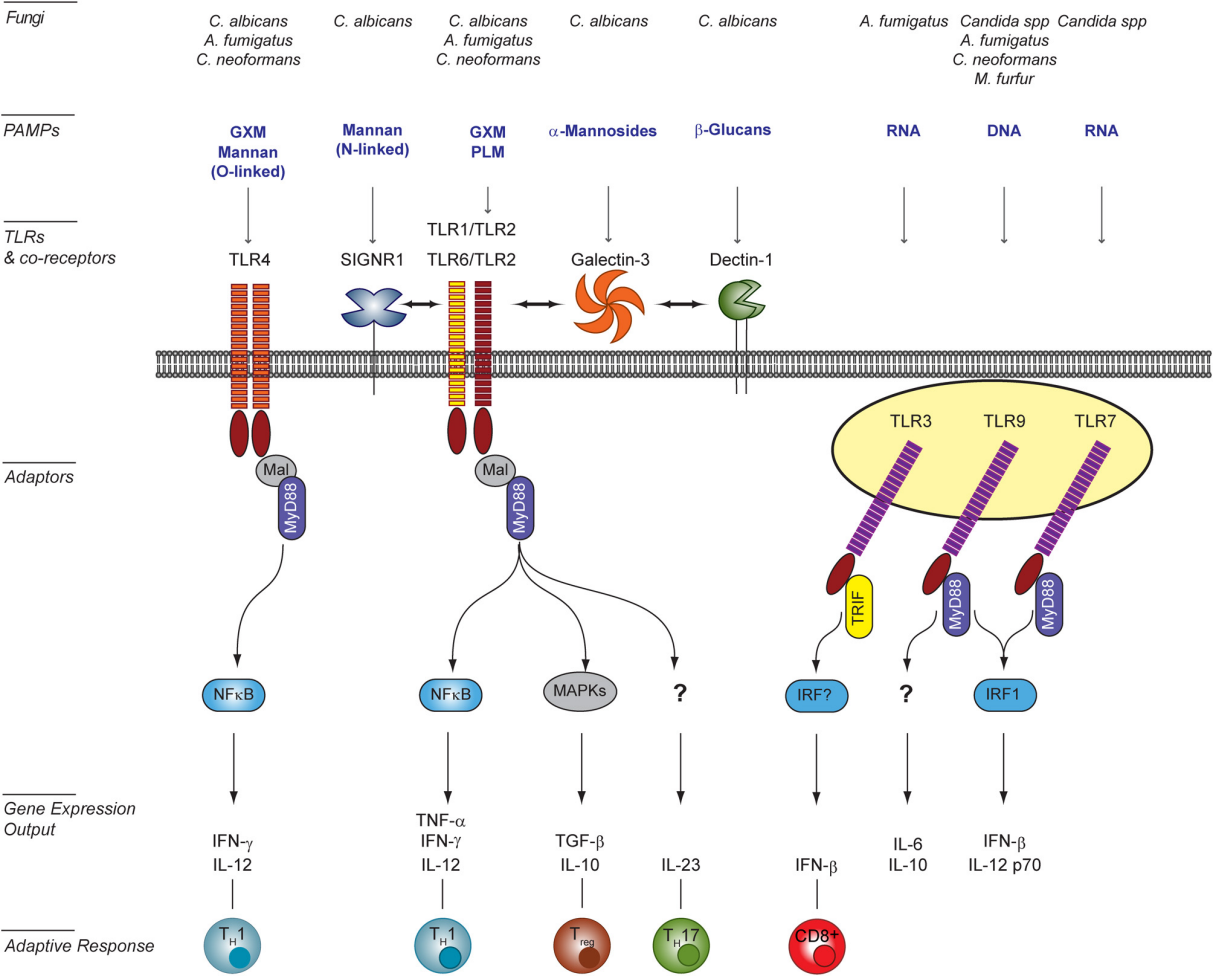
**TLR signaling induced in host cells upon interaction with fungal pathogens.** Surface Toll-like receptors (TLRs), as well as endosomal TLRs participate to the recognition of fungal PAMPs [e.g., O- and N-linked mannans, phospholipo-mannan (PLM), glucuronoxylomannan (GXM), α-mannosides, β-glucans, DNA, and RNA]. Activation of surface TLRs involves their homo- (TLR4) or hetero-dimerisation (TLR2/TLR1 or TLR6). The diversity of signaling pathways is increased by the involvement of co-receptors of the C-type lectin family (e.g., SIGNR1 and Dectin-1) or Galectin-3. Confirmed physical interactions between PRRs are represented by double-head arrows. The integration of simultaneously activated signaling pathways occurs at the level of intracellular adaptors and transcription factors shared between overlapping pathways. The resulting cytokine responses shape the activation of the adaptive response and ultimately modulate the outcome for the host. This figure was adapted from Bourgeois et al. (2010) by including newly published data from Biondo et al. (2012) and Takahara et al. (2012), and as reviewed in Romani (2011) and Leibundgut-Landmann et al. (2012). *A. fumigatus, Aspergillus fumigatus; C. albicans, Candida albicans; Candida spp, Candida* species; *C. neoformans; Cryptococcus neoformans; M. furfur, Malassesia furfur*.

The precise molecular nature of fungal PAMPs that active specific TLRs is difficult to pin down due to the often collaborative mechanism of TLR recognition and the plasticity of the fungal cell wall. Whereas fungal PAMPs are clearly recognized by a number of TLRs (i.e., TLR2/1, TLR4, TLR3, TLR2/6, TLR7, and TLR9), they are not the primary receptors driving pathogen engulfment. Fungal PAMPs for cell-surface TLRs have been mainly characterized for *Candida albicans*, but they remain mostly unknown for other fungi. For *C. albicans*, mutants with specific cell wall defects have facilitated the identification of PAMPs. Because cell-wall mutations often attenuate virulence or induce compensatory alteration of the cell wall composition (Murciano et al., 2011), altered immune responses to such mutants should be interpreted with caution. Nonetheless these studies have proven useful in identifying cell wall components activating TLRs (Figure 1). For example, TLR2 recognizes fungal β-glucans of several fungal species (Viriyakosol et al., 2005; Netea et al., 2006; Sorgi et al., 2009). In addition, it also specifically interacts with phospholipo-mannans (PLMs), linear beta-1,2-oligomannoside structures that are unique to *C. albicans* (Jouault et al., 2003). TLR2 is also stimulated by as yet unidentified ligands present on conidia and hyphae forms of *A. fumigatus* (Netea et al., 2003). TLR2/TLR1 and TLR2/TLR6 heterodimers are receptors for the glucuronoxylomannan (GXM) component of *Cryptococcus neoformans* (Fonseca et al., 2010). Notably, *A. fumigatus* activates mouse but not human TLR2/6 heterodimers, whereas TLR2/1 heterodimers recognize *A. fumigatus* both in human and mice (Rubino et al., 2012). This is a striking example of differences between human and mice in fungal recognition. TLR4 is activated upon ligation of *C. albicans* O-linked mannans (Netea et al., 2006), as well as *C. neoformans* GXM (Shoham et al., 2001). Ligands for TLR4 are present as well on *A. fumigatus* conidia but not hyphae (Netea et al., 2003).

In addition, to cell surface PAMPs, nucleic acids liberated from fungi within the phagosome also stimulate or modulate the dynamic host response during infection. TLR3 is activated by double-stranded RNA from *A. fumigatus* conidia in lung epithelial cells (Beisswenger et al., 2012). Single-stranded RNA from *Candida* spp. are ligands for TLR7 in mouse bone-marrow dendritic cells (BM-DCs) (Biondo et al., 2011). TLR9-mediated sensing of fungal genomic DNA (gDNA) appears conserved across fungal species (Nakamura et al., 2008; Ramirez-Ortiz et al., 2008; Miyazato et al., 2009; Biondo et al., 2011) and the recruitment of TLR9 to fungi-containing phagosome is observed with several fungal species (Kasperkovitz et al., 2011). Recognition of gDNA from *A. fumigatus* and *C. neoformans* occurs at unmethylated CpG motifs (Nakamura et al., 2008; Ramirez-Ortiz et al., 2008; Tanaka et al., 2011). By contrast, TLR9 detection of Candida gDNA does not seem to be restricted to these motifs (Miyazato et al., 2009).

### TLRs and modulation of immunity to fungi

#### Hematopoietic stem cells

Recent advances in hematopoietic stem cell (HSC) research suggest that commensal microbes, including fungi, “shape” the steady-state hematopoiesis through their interaction with TLRs (Boiko and Borghesi, 2012). Ligation of TLRs on mouse or human HSCs by microbial PAMPs affects both proliferation and differentiation (Baldridge et al., 2011; Boiko and Borghesi, 2012). At steady state, bone marrow from mice lacking TLR4, TLR9, or MyD88 exhibit enhanced reconstitution activity (Massberg and Von Andrian, 2009). Furthermore, in Drosophila, mutations in the *Toll/cactus* pathway cause a deregulated hematopoiesis (Qiu et al., 1998). Thus, TLR signaling in HSCs may serve two purposes: (1) it participates in the maintenance of basal hematopoietic homeostasis in the absence of triggers, and (2) it activates emergency hematopoiesis upon microbial infections. In a mouse model of systemic infection, *C. albicans* stimulates both proliferation and differentiation of HSCs and committed progenitors, driving enhanced granulopoiesis independently of G-CSF (Basu et al., 2000) but in a MyD88/TLR2-dependent fashion (Yanez et al., 2009, 2010, 2011). Notably, TLR2 promotes the differentiation of HSCs into macrophages and monocyte-derived DCs upon interaction with *Candida* spp. (Yanez et al., 2010, 2011).

#### Innate, adaptive and non-hematopoietic effector cells

Professional phagocytes such as neutrophils, monocyte/macrophages, and dendritic cells, are rapidly recruited at the site of infection upon fungal challenge (Lionakis et al., 2010, 2012; Majer et al., 2012; Wuthrich et al., 2012b). Notably, the lack of TLR2 impairs the early recruitment as well as killing capacity of neutrophils against *A. fumigatus* (Meier et al., 2003; Bellocchio et al., 2004a). Similarly, fewer neutrophil/monocytes are recruited in TLR2^−/−^ mice in comparison to wild-type animals at day 1 after post-peritoneal infection with live *C. albicans* (Tessarolli et al., 2010). Interestingly, upon intraperitoneal challenge with heat-killed *C. albicans*, TLR2 defficiency has no effect on early (4 h) phagocyte recruitment, but results in an enhanced macrophage recruitment in mutant versus control mice at day 3 after infection (Netea et al., 2004). These results suggest that TLR2 differentially modulates phagocyte recruitment during the course of candidiasis. Additionally, the use of live versus heat-killed *Candida* cells may affect both kinetics and nature of recruited phagocytes. Phagocytes emerging at day 1 of post-peritoneal infection with live *Candida* exhibit impaired nitric-oxide release, myeloperoxidase activity, chemokine, and cytokine production, as well as neutrophil survival in the absence of TLR2 (Tessarolli et al., 2010). Thyoglycolate-elicited TLR2^−/−^ neutrophils and macrophages show reduced phagocytic activity toward *C. albicans* than their wild-type counterparts (Tessarolli et al., 2010). Notably, no significant effects of TLR2 on phagocytosis by similar cells has also been reported (Netea et al., 2004), perhaps due to distinct experimental conditions used in the phagocyte preparations.

By contrast, the absence of TLR4 diminishes neutrophil effector functions against both *A. fumigatus* and *C. albicans* (Meier et al., 2003; Bellocchio et al., 2004b; Gasparoto et al., 2010), whereas TLR9 deficiency enhances the fungicidal capacity of neutrophils as well as macrophages (Bellocchio et al., 2004b; Kasperkovitz et al., 2011). Lack of TLRs also modulates the cytokine response in macrophages and dendritic cells upon fungal encounters (for review see, Romani, 2011) (Figure 1). In BM-DCs, but not in bone-marrow derived-macrophages, both TLR7 and TLR9 trigger the release of IFN-β in response to *Candida* (Biondo et al., 2011, 2012; Bourgeois et al., 2011). Notably, IL-12 p70 release is also dependent on both TLR7 and TLR9 in these cells (Biondo et al., 2012).

Th1 and Th17 are the principal Th subsets that contribute to a protective adaptive response to fungal pathogens (for review see, Hernandez-Santos and Gaffen, 2012; Leibundgut-Landmann et al., 2012; Wuthrich et al., 2012a). However, IL-17 and Th17 cells have been reported to be detrimental in certain mouse models of fungal infection (Zelante et al., 2009). In humans, by contrast, a defect in IL-17 signaling is linked to increased susceptibility to mucocutaneous Candida infection (Puel et al., 2011). Regulatory T-cells (Tregs) modulate the Th1/Th17 balance either by preventing expansion of the Th17 subset or by minimizing host damage (Loures et al., 2009). However, Tregs may also enhance the Th17 response and promote fungal clearance (Pandiyan et al., 2011). TLRs could influence the adaptive response either indirectly via activation of antigen presenting cells (APCs) or, by acting as co-receptors for TCRs directly on T-cells (for review see Jin et al., 2012). It is generally accepted that TLRs mediate the development of antifungal Th1 response. Notably, MyD88 is dispensable for the CD4^+^ T-cell priming against and trafficking during *Aspergillus* airway infections (Rivera et al., 2006), but it is required for the differentiation of fungi-specific CD4^+^ T-cells into IFN-γ-producing cells in lungs. TLR2, however, may promote T-reg differentiation. Indeed, TLR2^−/−^ mice have reduced levels of natural Tregs in comparison with wild-type mice, suggesting that TLR2 also regulates Treg homeostasis. In a *Paracoccidoides brasiliensis* intratracheal infection model, TLR2 promotes Treg expansion, thereby limiting Th17 cell differentiation and tissue pathology (Loures et al., 2009). By contrast, in a systemic infection model of candidiasis, TLR2-mediated recognition of *Candida* triggers IL-10 production and decreases Th1 polarisation (Netea et al., 2004). Recent studies suggest that TLRs may also play a direct role in the induction of a Th17 antifungal response: TLR6 exerts protective effects in a model of chronic *Aspergillus*-induced asthma, by promoting IL-23 release and a subsequent Th17 response (Moreira et al., 2011). In a skin-resident DC subset, Langerhans cells, MyD88 is required for their full activation and function in response to *C. albicans* infection, including the development of a Th17 response (Haley et al., 2012). Similarly, in a *Blastomyces dermatidis*-specific TCR mouse model, MyD88 but not dectin-1 is required for the development of a vaccine-induced Th17 subset and resistance to infection, which is consistent with the involvement of TLRs in DC activation (Wuthrich et al., 2011). Furthermore, TLR3-defficient mice fail to activate protective memory-CD8^+^ T cells following vaccination by *A. fumigatus* (Carvalho et al., 2012b). Thus, some TLRs expressed in APCs may be good candidates to stimulate DCs for antifungal vaccination strategies both as danger signals and to condition professional APCs to induce the appropriate class of protective adaptive immunity (for review see, Iannitti et al., 2012; Roy and Klein, 2012).

Terminally differentiated epithelial cells also take an active part in antifungal defense and immune surveillance (for review see, Naglik and Moyes, 2011; Weindl et al., 2011). The TLR expression levels are altered in these cells upon fungal infection, and their cytokine response is, at least in part, TLR-dependent. In mice, TLR4 is required for protection of epithelial cells from fungal invasion in the presence of polymorphonuclear leucocytes (PMNs) (Weindl et al., 2007). Similarly, TRIF^−/−^epithelial cells are more susceptible to *A. fumigatus* due to over activation of Th17 cytokines and down-regulation of Th1-Tregs (De Luca et al., 2010). Thus, TLR signaling in epithelial cells may modulate the inter-cellular communication and cooperation between hematopoietic and non-hematopoietic cells. Deregulation of underlying processes can enhance immunopathology and impair clearance (De Luca et al., 2010). Notably, TLRs also modulate the ability of epithelial cells and innate immune cells to sense and respond to danger signals others than established TLR ligands, including host or fungal proteases or other host “damage-associated molecular patterns” (DAMPs) (Moretti et al., 2008; Sorci et al., 2011).

## Mechanisms modulating toll-like receptor signaling during fungal recognition

Because microbial pathogens usually carry multiple classes of PAMPs, their recognition may involve the simultaneous or sequential activation of several PRRs from different families. Collaboration between PRRs and/or cross talk between their signaling pathways can enhance the specificity and coverage of PAMP recognition and enables a tailored host response (Van De Veerdonk et al., 2008a) (Figure 1). TLR2 transduces signals as a heterodimer recruiting either, TLR1 or TLR6 (Ozinsky et al., 2000). However, the functional consequences of these TLR cooperations for fungal recognition remain ill-defined. In addition, several molecules including C-type lectins or other carbohydrate-binding proteins have been identified as TLR2 co-receptors (e.g., Dectin-1, SIGNR1, and Galectin-3). Interestingly, depending on the co-receptor involved, co-ligation of TLR2 may either enhance a TLR2-dependent response (Smeekens et al., 2010; Takahara et al., 2012) or modulate its PAMPs specificity (Jouault et al., 2006). Dectin-1 has also been shown to synergies with TLR4 signaling (Ferwerda et al., 2008).

The molecular basis of signaling pathway crosstalk is just beginning to be investigated (reviewed in Hontelez et al., 2012). Dectin-1 signaling requires its clustering and the formation of a phagocytic synapse (Goodridge et al., 2011). No physical interaction between TLR2 and Dectin-1 have been reported so far, but TLR2 co-immunoprecipitates with Galectin-3 following stimulation with *C. albicans* (Jouault et al., 2006). Interestingly, Galectin-3 also co-immunoprecipitates with Dectin-1 (Esteban et al., 2011), suggesting that Galectin-3 may mediate the cooperation between TLR2 and Dectin-1 signaling. TLR2 also co-immunoprecipitates with SIGNR1 (Takahara et al., 2012). Thus, the dynamic clustering and/or exclusion of PRRs from the phagocytic synapse may control and modulate signaling cross talks during the initial immune response to surface PAMPs. Subsequent liberation of fungal PAMPs such as nucleic acids, through fungal pathogen degradation in the course of phagosome maturation may promote further recruitment of PRRs (Stuart and Ezekowitz, 2005; Kasperkovitz et al., 2010).

In addition to microbial PAMPs, host DAMPs arising from tissue damage such as S100B proteins are also released at the site of inflammation during infection. In a TLR2-dependent fashion, low doses of S100B proteins promote fungal clearance and protect against inflammation-induced epithelial damage in lungs of mice with *A. fumigatus* intranasal infections. By contrast, the TLR3/TRIF axis may reduce over-production of S100B proteins, thereby preventing exacerbation of the inflammation reaction to promote its resolution (Sorci et al., 2011). Thus, host DAMPs may collaborate with PAMP-activated TLRs to control the outcome of the inflammatory response.

*C. albicans* is uniquely recognized by TLR2 after antifungal treatment that targets and alters the cell wall (Roeder et al., 2004). Similarly, pretreatment of *C. albicans* or *A. fumigatus* with anti-fungal drugs enhance their ability to stimulate TLR expression in human PMNs (Salvenmoser et al., 2010). These results suggest that beside their direct fungicidal properties, antimycotics may also facilitate pathogen detection by the host and thereby, facilitate clearance.

## Fungal strategies to escape or subvert detection by toll-like receptors

Many fungal pathogens have developed strategies to escape or subvert host immune recognition systems, including sensing by TLRs or other PRRs (for review see, Collette and Lorenz, 2011). Cell wall remodeling upon environmental stress or during hyphae formation may change PAMP composition and alter accessibility for TLRs as observed for many species (Hohl et al., 2005; Collette and Lorenz, 2011). Furthermore, formation of large cellular structures either by germination and filamentation in dimorphic fungi, or by nuclear replication without fission in *Cryptococcus*, can hamper phagocytosis (Okagaki et al., 2010; Zaragoza et al., 2010) and thereby, is likely to prevent activation of the intra-phagosomal recognition processes.

*Cryptococcus* spp. and several other fungi secrete polysaccharides and protein cargos through dedicated exosomes upon host interaction. Supernatant of *C. neoformans* cultures inhibit TLR9 activation by *C. neoformans* DNA (Yamamoto et al., 2011). Similarly, *A. fumigatus* cell wall components differentially modulate TLR2 and TLR4 signaling (Chai et al., 2011).

Activation of endosomal TLR7-9 is controlled by their timely recruitment to the phagosome and proteolytic processing upon ligand binding (Ewald and Barton, 2011). Thus, modulation of intracellular protein trafficking and phagosome maturation, are likely to influence the recognition of fungal pathogens by endosomal TLRs. *Candida* spp. as well as *H. capsulatum* prevent phagosomal maturation and acidification (Eissenberg et al., 1993; Marcil et al., 2008; Fernandez-Arenas et al., 2009; Garcia-Rodas et al., 2011; Seider et al., 2011). *C. neoformans* and *C. albicans* share the ability to escape the phagosome, although using entirely distinct mechanisms (Collette and Lorenz, 2011). Paradoxically, the rapid recruitment of TLR9 to the fungus-containing phagosomes favors persistence, suggesting that this receptor may be exploited as an immune evasion strategy by several fungal species (Kasperkovitz et al., 2010, 2011).

## TLR-signaling and inborn susceptibility to fungal infections

### Mouse models of fungal infections

Mice lacking MyD88, the signaling adaptor shared by several surface and endosomal receptors, but also by the IL-1, IL-18, and IL-33 receptors, are hypersensitive to systemic *C. albicans* infections (Bellocchio et al., 2004a; Villamon et al., 2004), as well as to intraperitoneal and intranasal *C. neoformans* infections (Yauch et al., 2004; Biondo et al., 2005). Fungal clearance is impaired in MyD88^−/−^ mice during systemic and intra-gastric candidiasis, pulmonary, as well as corneal aspergillosis, and during *C. neoformans* infections (Bellocchio et al., 2004a; Yauch et al., 2004; Biondo et al., 2005; De Luca, 2007; Leal et al., 2010). Consistently, expression of Th1 and inflammatory cytokines during *C. neoformans* infections is lower in mice lacking MyD88 when compared to wild-type mice (Biondo et al., 2005).

In corneal aspergillosis, TRIF-deficient mice do not exhibit a fungal killing defect (Leal et al., 2010). However, in intra-gastric infection models, TRIF^−/−^ mice fail to prevent spreading of *C. albicans* to peripheral organs (De Luca, 2007). In pulmonary aspergillosis, TRIF^−/−^ mice as well as TLR3^−/−^ mice are highly susceptible to infection and develop pathogen-induced inflammation (De Luca et al., 2010; Carvalho et al., 2012b). Thus, these data suggest that TLR signaling adaptors drive pathways with distinct effector functions in fungal pathogenesis. TRIF pathways appear to promote tolerance, whereas MyD88 is required for fungicidal activity (Romani, 2011).

Mice lacking TLR2 exhibit an intrinsic defect in the number of CD4^+^CD25^+^Treg subset that maintains peripheral tolerance, but may also dampen the immune response to infection (Netea et al., 2004). Upon intravenous *Candida* infection, absence of Tregs results in improved fungal clearance 7 days after infection and better survival of TLR2^−/−^ mice when compared to wild-type mice (Bellocchio et al., 2004a; Netea et al., 2004). By contrast, in an intraperitoneal model of candidiasis, clearance is impaired 1 day afterinfection in the absence of TLR2 (Tessarolli et al., 2010). TLR2-deficient mice infected with *P. jirovecii* display intenser severity in symptoms, as well as increased fungal burden and decreased TNFα and nitric oxide release in the lungs (Wang et al., 2008).

Immunosuppressed TLR2^−/−^ mice (neutropenic and treated with antibiotics) have an increased susceptibility to *A. fumigatus* following intratracheal infection and increased fungal burden in the lung in comparison to wild-type immunosuppressed animals (Balloy et al., 2005). By contrast, cyclophosphamide-treated TLR2^−/−^mice are equally susceptible to intranasal *Aspergillus* infections than control mice, although untreated mutant mice have higher lung fungal burden (Bellocchio et al., 2004a). In a model of chronic fungal asthma, TLR2^−/−^ mice show impaired airway hyper-responsiveness to *A. fumigatus* and reduced fungal clearance at early infection stages. As a result of fungal persistence, but also perhaps due to the deficiency in the Treg subset, airway hyper-responsiveness increases during the adaptive phase of this disease model (Buckland et al., 2008). In a model of mouse corneal inflammation, TLR2-deficiency does not affect immune cell infiltration or fungal clearance (Leal et al., 2010). Conversely, *Aspergillus*-induced corneal inflammation in rats is decreased following application of TLR2 siRNA when compared to non-specific siRNA and fungal clearance, as well as the outcome of fungal disease, are improved (Guo et al., 2012).

TLR2^−/−^ mice are also more susceptible to intranasal or intraperitoneal cryptococcal infections and exhibit higher fungal burden (Yauch et al., 2004; Biondo et al., 2005), as well as decreased inflammatory cytokines (Biondo et al., 2005). By contrast, in a model of *Cryptococcus* intratracheal infections, TLR2^−/−^ mice show no changes in survival in comparison to control mice (Nakamura et al., 2006). Thus, in most mouse models of fungal airway infection, TLR2 appears to contribute to fungal clearance, perhaps by modulating the induction of inflammatory cytokines. However, depending on the airway infection model, the disease outcome itself may vary from unaffected to increased susceptibility. Conversely, in systemic or corneal infection models, T-regs may hinder fungal clearance and worsen the outcome of fungal disease.

Mice lacking TLR4 are either hyperresistant, hypersensitive or equally susceptible to fungal challenges than wild-type mice, depending on the *C. albicans* or the mouse strains used or the infection route. The recently described variable recognition of different *C. albicans* strains by TLR4 may account for some of these apparent discrepancies (Netea et al., 2010). TLR4 recognition may be required to elicit host defense only against strains inducing proinflammatory cytokines in a TLR4-dependent fashion. Interestingly, *C. albicans* mutants lacking particular glycosylation patterns such as O-glycosylation, are specifically recognized by TLR4, and lead to enhanced activation of macrophages (Lewis et al., 2012). Hence, intraspecies variability in cell wall glycosylation can determine the nature of the interaction between TLR4 and *C. albicans* strains, and thereby the type and intensity of the host immune response. A similar mechanism may be operating in flies, since Toll-signaling in *Drosophila* can distinguish virulent from avirulent *Candida* strains (Glittenberg et al., 2011).

Lack of TLR4 exacerbates the host inflammatory response to *P. jirovecii* in mice, though without affecting pathogen clearance (Ding et al., 2005). However, the course of intranasal, tracheal, intravenous, or intraperitoneal infections with *C. neoformans* remains unaffected in these mutant mice in comparison with control mice (Yauch et al., 2004; Biondo et al., 2005; Nakamura et al., 2006). In a model of pulmonary fungal infection with *C. posadasii*, lack of TLR4 improved fungal clearance (Awasthi, 2010). By contrast, Aspergillus killing is impaired in TLR4^−/−^ mice in a model of corneal inflammation, although immune cell infiltration is unaffected (Leal et al., 2010). Whether intraspecies variabilities in cell wall composition can determine the nature of interactions between TLR4 and other fungal species than *Candida* remains open.

TLR3-defficient mice fail to activate protective memory-CD8^+^ T cells following *Aspergillus* vaccination (Carvalho et al., 2012b). Mice deficient in TLR7 are more susceptible to systemic infections by low doses of *C. albicans* than their WT wild-type littermates. However, when challenged with higher doses, the mutant mice are equally susceptible to infection than the control mice (Biondo et al., 2012).

A lack of TLR9 impairs clearance and decreases survival to *C. neoformans* challenge in an intranasal infection model (Nakamura et al., 2008; Wang et al., 2011). In this model, TLR9 contributes to the early induction of CCL7 and IFN-γ, thereby promoting recruitment and activation of DCs and other effector cells. Similarly, a lack of TLR9 during intratracheal *C. neoformans* infections results in impaired clearance during the adaptive phase, decreased recruitment of lymphocyte and macrophages, as well as alternative activation of macrophages (Zhang et al., 2010). By contrast, TLR9^−/−^ mice depleted from neutrophils/inflammatory monocytes prior to tracheal infection with *A. fumigatus*, exhibit delayed mortality in comparison to depleted control mice (Ramaprakash et al., 2009). Notably, although TLR9-deficiency in immunosuppressed mice has no effect on survival to *A. fumigatus* after intranasal infections, it improves clearance (Bellocchio et al., 2004a).

Using high dose challenges, TLR9^−/−^ mice show no significant alterations in survival to clinical isolates of *C. albicans* (Van De Veerdonk et al., 2008b; Miyazato et al., 2009; Biondo et al., 2012), and even enhanced clearance when infected with an avirulent strain (Bellocchio et al., 2004a). However, a lower fungal dose leads to increased susceptibility to systemic candidiasis and impaired fungal clearance in TLR9^−/−^ mice (Biondo et al., 2012), indicating that the fungal load may determine the role of TLR9 during infection. In summary, TLR9 signaling appears to mediate clearance of *Cryptococcus* and low doses of *C. albicans*. However, TLR9 may be exploited for immune evasion by *C. albicans* at higher doses or *A. fumigatus*. Interestingly, endosomal TLR7, TLR8, and TLR9 show inhibitory cooperations or interactions (Wang et al., 2006). Notably, autoimmune models have been instrumental for a better understanding of regulatory interactions between endosomal TLRs, indicating a modulatory role of TLR9 on TLR7 signaling (Ewald and Barton, 2011). Thus, the improved clearance observed in TLR9^−/−^ phagocytes may result from hyperactivated TLR7 signaling in absence of TLR9 (Nickerson et al., 2010).

Thus, contrary to the strong impact of MyD88 deficiency on fungal clearance and disease susceptibility, data from animal models with single TLR defects are more difficult to interpret or often even conflicting. This may result, at least in part, from the central role of MyD88 as adapter protein not only for most TLRs but also for cytokine receptors recognizing IL-1, IL-18, and IL33. IL-1R signaling for instance has an essential function in the defense against *C. albicans* but not all fungal species (Bellocchio et al., 2004a; Leal et al., 2010; Wang et al., 2011). Hence, the contribution of individual TLRs to protection against infection appears to greatly vary depending on the fungal strain and/or species, infection model, infection dose as well as the genetics of mouse strains.

Furthermore, unchanged susceptibility of some TLR-deficient mouse strains may be due to a possible dual role of TLRs on pathogen clearance but also host tolerance (Ayres and Schneider, 2008; Carvalho et al., 2012a; Medzhitov et al., 2012). In this scenario, lack of a given TLR may impair fungicidal mechanisms, but also improve host tolerance to infection such that the final outcome of disease appears unaffected by the TLR absence. The notion that tolerance to infections is as crucial for resolution of infections as it is for resistance to the pathogen is a relatively recent conceptual idea in mammalian infection biology (Schneider and Ayres, 2008; Carvalho et al., 2012a; Medzhitov et al., 2012). Consequently, experimental approaches that enable identification and quantification of trade-offs between tolerance and resistance may help to better characterize and understand the roles and contributions of TLRs to microbial infections in general.

### In humans

By sharp contrast to mice, humans with MyD88 signaling defects do not have increased incidence of fungal infections (Von Bernuth et al., 2008). This may relate to the fact that *Candida* spp. are commensal colonizers of humans but not of mice (Savage and Dubos, 1967), but also that significant differences exist between human and mouse TLR signaling (Rehli, 2002). However, some TLR single nucleotide polymorphisms (SNPs) in TLR genes significantly augment the risk of contracting fungal infection in humans (summarized in Table 1). TLR1 SNPs are associated with higher susceptibility to candidemia in humans. In agreement, cytokine release by blood monocytes in response to *C. albicans* is impaired in these patients (Plantinga et al., 2012). Invasive aspergillosis is one of the most important nosocomial infections after HSC transplantations (Cunha et al., 2011; Lamoth et al., 2011). Recipients of allogeneic HSC transplants carrying the TLR1 Arg80Thr or both TLR1 Asn248Ser and TLR6 Ser249Pro SNPs are more prone to *Aspergillus* infections (Kesh et al., 2005). Increased susceptibility to aspergillosis is also observed in this “at-risk” group in patients carrying a TLR3 +95C/A, but not a TLR3 L412F, SNP (Carvalho et al., 2012b). As a result of the TLR3 +95C/A SNP, activation of a memory-protective CD8+ T-cell responses against *Aspergillus* is impaired (Carvalho et al., 2012b). By contrast, TLR3 L412F SNP is associated with increased prevalence of cutaneous candidiasis and impaired TLR3 signaling (Nahum et al., 2011, 2012).

**Table 1:** **TLR polymorphisms associated with increased susceptibility to fungal diseases**.

**Gene**	**SNPs or haplotypes**	**Effect**	**Disease**	**Outcome**	**References**
**TLR1**	R80T, N248S, I602S	Reduced cytokine production by PBMCs *in vitro*	Invasive aspergillosis, *C. albicans* systemic infections	Susceptibility	Kesh et al., 2005; Plantinga et al., 2012
**TLR3**	+95C/A	Failure to activate CD8^+^ T-cell response	Invasive aspergillosis	Susceptibility	Carvalho et al., 2012b
	L412F	Decreased TLR3 functionality	Chronic mucocutaneous candidiasis	Susceptibility	Nahum et al., 2011
**TLR4**	D299G/T399I	Predicted to impair ligand binding	Invasive aspergillosis, *A. fumigatus*, CCPA, *C. albicans* systemic infections	Susceptibility	Van Der Graaf et al., 2006; Bochud et al., 2008; Carvalho et al., 2008; De Boer et al., 2011
**TLR6**	S249P	Unknown	Invasive aspergillosis	Susceptibility	Kesh et al., 2005
**TLR9**	T-1237C	Increased NF-κB binding affinity	ABPA	Susceptibility	Carvalho et al., 2008

ABPA, allergic bronchopulmonaryaspergillosis; CCPA, chronic cavitary pulmonary aspergillosis; C. albicans, Candida albicans; A. fumigatus, Aspergillus fumigatus. Modified from Romani, (2011)

An enhanced risk of chronic pulmonary aspergillosis has been linked to allele G on TLR4 Asp299Gly (Carvalho et al., 2008). The prevalence of this SNP in association with TLR4 Thr399Ile was also higher in a patient cohort suffering from Candida blood-stream infection in comparison to the control group (Van Der Graaf et al., 2006). Peripheral blood mononuclear cells (PBMCs) from patients carrying both polymorphisms exhibited enhanced IL-10 release upon *C. albicans* challenge (Van Der Graaf et al., 2006) but not PBMCs from patients carrying only the TLR4 Asp299Gly (Van Der Graaf et al., 2005). Finally, allele C on TLR9 T-1237C has been linked to a higher susceptibility to allergic bronchopulmonary aspergillosis (Carvalho et al., 2008). Hence, a growing body of evidence indicates that TLRs are actively involved in *Candida* and *Aspergillus* recognition in humans and most likely in recognition of other fungal pathogens as well. The challenges associated with such studies are the low number of patients/groups and the relative low risk of infection in people carrying these SNPs, perhaps due to genetic redundancy in certain components (Netea et al., 2012) or because of TLR dual roles in shaping both resistance and tolerance to fungi. The consequences of SNPs may become more obvious in individuals with a weakened immune system. The continuous identification of new SNPs and the characterization of their effects at the molecular and cellular level will help a further uncovering of TLR roles in the antifungal immune response in humans. The identification of functional SNPs may also serve to detect “at-risk” patients and design efficient prophilaxy when necessary. Interestingly, age-related alterations in the host response to fungi may also occur, as recent data indicate that neutrophils from elderly individuals express lower levels of TLR2 than younger patients (Gasparoto et al., 2012).

## Conclusions and perspectives

Historically, TLRs were the first described specific PRRs for fungal pathogens. The past 12 years of research on host immune response to fungi have delineated the roles of several TLRs in mediating cytokine response upon fungal interaction. Importantly, mouse survival studies have uncovered somewhat contradictory (protective or detrimental) or even non-conclusive (without effect) data on the role of TLRs in the murine antifungal response. Similarly, humans lacking MyD88, an ubiquitous signaling adaptor for TLRs, fail to show increased incidences of fungal infections. To date, no genetic defects in human TLRs have been associated with a primary immune deficiency conferring increased susceptibility to either mucocutaneous or invasive fungal infections (reviewed in Lilic, 2012). However, certain TLR SNPs are clearly associated with increased susceptibility to fungal disease in specific “at-risk” populations, suggesting that TLRs are involved in fine-tuning the outcome of various host-fungus interactions (e.g., commensalism, symbiosis, latency, infection, and dissemination). In agreement, at the cellular levels, most TLRs appear not to be required for the primary step of sensing and engulfment of fungal microbes by innate immune cells. However, TLRs are recruited to sites of host cell-microbe recognition and modulate the subsequent host-fungi interplay in maturing phagosomes. Furthermore, certain TLRs mediate specific protective adaptive responses. Thus, such TLRs may be suitable targets for activating DCs in efforts to generate fungal vaccines (Iannitti et al., 2012; Roy and Klein, 2012).

The outcome of a host response is determined by several phases, including activation of inflammatory defenses aimed at eliminating pathogens. However, uncontrolled host inflammatory responses promote sepsis and can be fatal for the host (Lionakis et al., 2012; Majer et al., 2012). Thus, the ability of the host to (1) control the inflammatory response in a timely manner and (2) to activate tissue repair mechanisms to resolve organ damages are critical components of a proper host immune response (Medzhitov et al., 2012). In this prospect, new findings suggest that TLRs and other PRRs may be involved in epithelial resistance to fungi (Weindl et al., 2011; Iliev et al., 2012). Whether TLR signaling also modulates the activity of professional phagocytes that promote the resolution of inflammation and repair processes during fungal infection (Sica and Mantovani, 2012), remains to be established.

Finally, exciting recent advances in the molecular mechanisms driving an immune memory of innate origin (Netea et al., 2011), open new fields concerning possible roles of TLRs in host-fungus interactions. Indeed, fungal β-glucans acting through Dectin-1 are clearly able to prime mouse and human monocytes to elicit a stronger inflammatory response upon restimulation with *C. albicans* or other PAMPs, and this by inducing chromatin remodeling (Quintin et al., 2012). The effect of β-glucans, reminiscent of the LPS-mediated priming of some TLR4-induced genes through chromatin modifications (Foster and Medzhitov, 2009), raises the question whether other fungal PAMPs, such as nucleic acids, may also induce monocyte priming in a TLR-dependent fashion. These data also suggest new ways by which PRRs in innate cells, including TLRs, exploit host chromatin remodeling to shape the host immune response to fungi at steady-state and during dynamics of infections (Tierney et al., 2012).

## Funding

This work was funded by the Christian Doppler Research Society and by a grant from the FWF-DACH programme to Karl Kuchler (FWF-Project API-746-B11). Christelle Bourgeois was also supported by the EC Marie Curie Training Network *“CanTrain”* (CT-2004-512481).

### Conflict of interest statement

The authors declare that the research was conducted in the absence of any commercial or financial relationships that could be construed as a potential conflict of interest.

## References

[B1] Awasthi S. (2010). Susceptibility of TLR4-defective C3H/HeJ mice to *Coccidioides posadasii* infection. Med. Mycol. 48, 470–475 10.3109/13693780903226019 20370361

[B2] Ayres J. S. Schneider D. S. (2008). A signaling protease required for melanization in Drosophila affects resistance and tolerance of infections. PLoS Biol. 6:e305 10.1371/journal.pbio.0060305 19071960 PMC2596860

[B3] Baldridge M. T. King K. Y. Goodell M. A. (2011). Inflammatory signals regulate hematopoietic stem cells. Trends Immunol. 32, 57–65 10.1016/j.it.2010.12.003 21233016 PMC3042730

[B4] Balloy V. Si-Tahar M. Takeuchi O. Philippe B. Nahori M. A. Tanguy M. (2005). Involvement of *toll*-like receptor 2 in experimental invasive pulmonary aspergillosis. Infect. Immun. 73, 5420–5425 10.1128/IAI.73.9.5420-5425.2005 16113258 PMC1231150

[B5] Basu S. Hodgson G. Zhang H. H. Katz M. Quilici C. Dunn A. R. (2000). “Emergency” granulopoiesis in G-CSF-deficient mice in response to *Candida albicans* infection. Blood 95, 3725–3733 10845903

[B6] Beisswenger C. Hess C. Bals R. (2012). *Aspergillus fumigatus* conidia induce interferon-beta signalling in respiratory epithelial cells. Eur. Respir. J. 39, 411–418 10.1183/09031936.00096110 21778165

[B7] Bellocchio S. Montagnoli C. Bozza S. Gaziano R. Rossi G. Mambula S. S. (2004a). The contribution of the *Toll*-like/IL-1 receptor superfamily to innate and adaptive immunity to fungal pathogens *in vivo*. J. Immunol. 172, 3059–3069 14978111 10.4049/jimmunol.172.5.3059

[B8] Bellocchio S. Moretti S. Perruccio K. Fallarino F. Bozza S. Montagnoli C. (2004b). TLRs govern neutrophil activity in aspergillosis. J. Immunol. 173, 7406–7415 15585866 10.4049/jimmunol.173.12.7406

[B9] Biondo C. Malara A. Costa A. Signorino G. Cardile F. Midiri A. (2012). Recognition of fungal RNA by TLR7 has a non-redundant role in host defense against experimental candidiasis. Eur. J. Immunol. 42, 2632–2643 10.1002/eji.201242532 22777843

[B10] Biondo C. Midiri A. Messina L. Tomasello F. Garufi G. Catania M. R. (2005). MyD88 and TLR2, but not TLR4, are required for host defense against *Cryptococcus neoformans*. Eur. J. Immunol. 35, 870–878 10.1002/eji.200425799 15714580

[B11] Biondo C. Signorino G. Costa A. Midiri A. Gerace E. Galbo R. (2011). Recognition of yeast nucleic acids triggers a host protective type I interferon response. Eur. J. Immunol. 41, 1969–1979 10.1002/eji.201141490 21480215

[B12] Bochud P. Y. Chien J. W. Marr K. A. Leisenring W. M. Upton A. Janer M. (2008). *Toll*-like receptor 4 polymorphisms and aspergillosis in stem-cell transplantation. N. Engl. J. Med. 359, 1766–1777 10.1056/NEJMoa0802629 18946062 PMC2656610

[B13] Boiko J. R. Borghesi L. (2012). Hematopoiesis sculpted by pathogens: *Toll*-like receptors and inflammatory mediators directly activate stem cells. Cytokine 57, 1–8 10.1016/j.cyto.2011.10.005 22079335 PMC3361504

[B14] Bourgeois C. Majer O. Frohner I. E. Lesiak-Markowicz I. Hildering K. S. Glaser W. (2011). Conventional dendritic cells mount a type I IFN response against *Candida* spp. requiring novel phagosomal TLR7-mediated IFN-β signaling. J. Immunol. 186, 3104–3112 10.4049/jimmunol.1002599 21282509

[B15] Bourgeois C. Majer O. Frohner I. E. Tierney L. Kuchler K. (2010). Fungal attacks on mammalian hosts: pathogen elimination requires sensing and tasting. Curr. Opin. Microbiol. 13, 401–408 10.1016/j.mib.2010.05.004 20538507

[B16] Brown G. D. Denning D. W. Levitz S. M. (2012). Tackling human fungal infections. Science 336, 647 10.1126/science.1222236 22582229

[B17] Buckland K. F. O'Connor E. Murray L. A. Hogaboam C. M. (2008). *Toll*-like receptor 2 modulates both innate and adaptive immune responses during chronic fungal asthma in mice. Inflamm. Res. 57, 379–387 10.1007/s00011-008-8004-y 18787777

[B18] Carvalho A. Cunha C. Bozza S. Moretti S. Massi-Benedetti C. Bistoni F. (2012a). Immunity and tolerance to fungi in hematopoietic transplantation: principles and perspectives. Front. Immunol. 3:156 10.3389/fimmu.2012.00156 22707953 PMC3374351

[B19] Carvalho A. De Luca A. Bozza S. Cunha C. D'Angelo C. Moretti S. (2012b). TLR3 essentially promotes protective class I-restricted memory CD8(+) T-cell responses to *Aspergillus fumigatus* in hematopoietic transplanted patients. Blood 119, 967–977 10.1182/blood-2011-06-362582 22147891

[B20] Carvalho A. Pasqualotto A. C. Pitzurra L. Romani L. Denning D. W. Rodrigues F. (2008). Polymorphisms in *toll*-like receptor genes and susceptibility to pulmonary aspergillosis. J. Infect. Dis. 197, 618–621 10.1086/526500 18275280

[B21] Chai L. Y. Vonk A. G. Kullberg B. J. Verweij P. E. Verschueren I. Van Der Meer J. W. (2011). *Aspergillus fumigatus* cell wall components differentially modulate host TLR2 and TLR4 responses. Microbes Infect. 13, 151–159 10.1016/j.micinf.2010.10.005 20971208

[B22] Collette J. R. Lorenz M. C. (2011). Mechanisms of immune evasion in fungal pathogens. Curr. Opin. Microbiol. 14, 668–675 10.1016/j.mib.2011.09.007 21955887

[B23] Cunha C. Rodrigues F. Zelante T. Aversa F. Romani L. Carvalho A. (2011). Genetic susceptibility to aspergillosis in allogeneic stem-cell transplantation. Med. Mycol. 49(Suppl. 1), S137–S143 10.3109/13693786.2010.508797 20718605

[B24] De Boer M. G. Jolink H. Halkes C. J. Van Der Heiden P. L. Kremer D. Falkenburg J. H. (2011). Influence of polymorphisms in innate immunity genes on susceptibility to invasive aspergillosis after stem cell transplantation. PLoS ONE 6:e18403 10.1371/journal.pone.0018403 21483748 PMC3070725

[B25] Del Poeta M. Chaturvedi V. (2012). Cryptococcus and cryptococcosis in the twenty-first century. Mycopathologia 173, 283–285 10.1007/s11046-012-9544-9 22531978

[B26] De Luca A. (2007). Functional yet balanced reactivity to *Candida albicans* requires TRIF, MyD88, and IDO-dependent inhibition of Rorc. J. Immunol. 179, 5999–6008 17947673 10.4049/jimmunol.179.9.5999

[B27] De Luca A. Bozza S. Zelante T. Zagarella S. D'Angelo C. Perruccio K. (2010). Non-hematopoietic cells contribute to protective tolerance to *Aspergillus fumigatus* via a TRIF pathway converging on IDO. Cell. Mol. Immunol. 7, 459–470 10.1038/cmi.2010.43 20835271 PMC4002959

[B28] Ding K. Shibui A. Wang Y. Takamoto M. Matsuguchi T. Sugane K. (2005). Impaired recognition by *Toll*-like receptor 4 is responsible for exacerbated murine *Pneumocystis pneumonia*. Microbes Infect. 7, 195–203 10.1016/j.micinf.2004.10.010 15725383

[B29] Eissenberg L. G. Goldman W. E. Schlesinger P. H. (1993). *Histoplasma capsulatum* modulates the acidification of phagolysosomes. J. Exp. Med. 177, 1605–1611 8496679 10.1084/jem.177.6.1605 PMC2191039

[B30] Esteban A. Popp M. W. Vyas V. K. Strijbis K. Ploegh H. L. Fink G. R. (2011). Fungal recognition is mediated by the association of dectin-1 and galectin-3 in macrophages. Proc. Natl. Acad. Sci. U.S.A. 108, 14270–14275 10.1073/pnas.1111415108 21825168 PMC3161568

[B31] Ewald S. E. Barton G. M. (2011). Nucleic acid sensing *Toll*-like receptors in autoimmunity. Curr. Opin. Immunol. 23, 3–9 10.1016/j.coi.2010.11.006 21146971 PMC3057394

[B32] Fernandez-Arenas E. Bleck C. K. Nombela C. Gil C. Griffiths G. Diez-Orejas R. (2009). *Candida albicans* actively modulates intracellular membrane trafficking in mouse macrophage phagosomes. Cell. Microbiol. 11, 560–589 10.1111/j.1462-5822.2008.01274.x 19134116

[B33] Ferwerda G. Meyer-Wentrup F. Kullberg B. J. Netea M. G. Adema G. J. (2008). Dectin-1 synergizes with TLR2 and TLR4 for cytokine production in human primary monocytes and macrophages. Cell. Microbiol. 10, 2058–2066 10.1111/j.1462-5822.2008.01188.x 18549457

[B34] Fonseca F. L. Nohara L. L. Cordero R. J. Frases S. Casadevall A. Almeida I. C. (2010). Immunomodulatory effects of serotype B glucuronoxylomannan from *Cryptococcus gattii* correlate with polysaccharide diameter. Infect. Immun. 78, 3861–3870 10.1128/IAI.00111-10 20547742 PMC2937472

[B35] Foster S. L. Medzhitov R. (2009). Gene-specific control of the TLR-induced inflammatory response. Clin. Immunol. 130, 7–15 10.1016/j.clim.2008.08.015 18964303 PMC3252731

[B36] Garcia-Rodas R. Gonzalez-Camacho F. Rodriguez-Tudela J. L. Cuenca-Estrella M. Zaragoza O. (2011). The interaction between *Candida krusei* and murine macrophages results in multiple outcomes, including intracellular survival and escape from killing. Infect. Immun. 79, 2136–2144 10.1128/IAI.00044-11 21422181 PMC3125833

[B37] Gasparoto T. H. De Oliveira C. E. Vieira N. A. Porto V. C. Gasparoto C. T. Campanelli A. P. (2012). The pattern recognition receptors expressed on neutrophils and the associated cytokine profile from different aged patients with Candida-related denture stomatitis. Exp. Gerontol. 47, 741–748 10.1016/j.exger.2012.07.003 22796226

[B38] Gasparoto T. H. Tessarolli V. Garlet T. P. Torres S. A. Garlet G. P. Da Silva J. S. (2010). Absence of functional TLR4 impairs response of macrophages after *Candida albicans* infection. Med. Mycol. 48, 1009–1017 10.3109/13693786.2010.481292 20465519

[B39] Glittenberg M. T. Silas S. MacCallum D. M. Gow N. A. Ligoxygakis P. (2011). Wild-type *Drosophila melanogaster* as an alternative model system for investigating the pathogenicity of *Candida albicans*. Dis. Model. Mech. 4, 504–514 10.1242/dmm.006619 21540241 PMC3124057

[B40] Goodridge H. S. Reyes C. N. Becker C. A. Katsumoto T. R. Ma J. Wolf A. J. (2011). Activation of the innate immune receptor Dectin-1 upon formation of a ‘phagocytic synapse’. Nature 472, 471–475 10.1038/nature10071 21525931 PMC3084546

[B41] Gow N. A. Hube B. (2012). Importance of the *Candida albicans* cell wall during commensalism and infection. Curr. Opin. Microbiol. 15, 406–412 10.1016/j.mib.2012.04.005 22609181

[B42] Gow N. A. Van De Veerdonk F. L. Brown A. J. Netea M. G. (2012). *Candida albicans* morphogenesis and host defence: discriminating invasion from colonization. Nat. Rev. Microbiol. 10, 112–122 10.1038/nrmicro2711 22158429 PMC3624162

[B43] Guo H. Gao J. Wu X. (2012). *Toll*-like receptor 2 siRNA suppresses corneal inflammation and attenuates *Aspergillus fumigatus* keratitis in rats. Immunol. Cell Biol. 90, 352–357 10.1038/icb.2011.49 21647173

[B44] Haley K. Igyarto B. Z. Ortner D. Bobr A. Kashem S. Schenten D. (2012). Langerhans cells require MyD88-dependent signals for *Candida albicans* response but not for contact hypersensitivity or migration. J. Immunol. 188, 4334–4339 10.4049/jimmunol.1102759 22442445 PMC3331889

[B45] Hernandez-Santos N. Gaffen S. L. (2012). Th17 cells in immunity to *Candida albicans*. Cell Host Microbe 11, 425–435 10.1016/j.chom.2012.04.008 22607796 PMC3358697

[B46] Hohl T. M. Van Epps H. L. Rivera A. Morgan L. A. Chen P. L. Feldmesser M. (2005). *Aspergillus fumigatus* triggers inflammatory responses by stage-specific beta-glucan display. PLoS Pathog. 1:e30 10.1371/journal.ppat.0010030 16304610 PMC1287910

[B47] Hontelez S. Sanecka A. Netea M. G. Van Spriel A. B. Adema G. J. (2012). Molecular view on PRR cross-talk in antifungal immunity. Cell. Microbiol. 14, 467–474 10.1111/j.1462-5822.2012.01748.x 22233321

[B48] Hube B. (2009). Fungal adaptation to the host environment. Curr. Opin. Microbiol. 12, 347–349 10.1016/j.mib.2009.06.009 19577508

[B49] Iannitti R. G. Carvalho A. Romani L. (2012). From memory to antifungal vaccine design. Trends Immunol. 33, 467–474 10.1016/j.it.2012.04.008 22647597

[B50] Iliev I. D. Funari V. A. Taylor K. D. Nguyen Q. Reyes C. N. Strom S. P. (2012). Interactions between commensal fungi and the C-type lectin receptor Dectin-1 influence colitis. Science 336, 1314–1317 10.1126/science.1221789 22674328 PMC3432565

[B51] Jin B. Sun T. Yu X. H. Yang Y. X. Yeo A. E. (2012). The effects of TLR activation on T-cell development and differentiation. Clin. Dev. Immunol. 2012, 836485 10.1155/2012/836485 22737174 PMC3376488

[B52] Jouault T. El Abed-El Behi M. Martinez-Esparza M. Breuilh L. Trinel P. A. Chamaillard M. (2006). Specific recognition of *Candida albicans* by macrophages requires galectin-3 to discriminate *Saccharomyces cerevisiae* and needs association with TLR2 for signaling. J. Immunol. 177, 4679–4687 16982907 10.4049/jimmunol.177.7.4679

[B53] Jouault T. Ibata-Ombetta S. Takeuchi O. Trinel P. A. Sacchetti P. Lefebvre P. (2003). *Candida albicans* phospholipomannan is sensed through *Toll*-like receptors. J. Infect. Dis. 188, 165–172 10.1086/375784 12825186

[B54] Kasperkovitz P. V. Cardenas M. L. Vyas J. M. (2010). TLR9 is actively recruited to *Aspergillus fumigatus* phagosomes and requires the N-terminal proteolytic cleavage domain for proper intracellular trafficking. J. Immunol. 185, 7614–7622 10.4049/jimmunol.1002760 21059889 PMC3600358

[B55] Kasperkovitz P. V. Khan N. S. Tam J. M. Mansour M. K. Davids P. J. Vyas J. M. (2011). *Toll*-like receptor 9 modulates macrophage antifungal effector function during innate recognition of *Candida albicans* and *Saccharomyces cerevisiae*. Infect. Immun. 79, 4858–4867 10.1128/IAI.05626-11 21947771 PMC3232661

[B56] Kesh S. Mensah N. Y. Peterlongo P. Jaffe D. Hsu K. VAN DEN Brink M. (2005). TLR1 and TLR6 polymorphisms are associated with susceptibility to invasive aspergillosis after allogeneic stem cell transplantation. Ann. N.Y. Acad. Sci. 1062, 95–103 10.1196/annals.1358.012 16461792

[B57] Lamoth F. Rubino I. Bochud P. Y. (2011). Immunogenetics of invasive aspergillosis. Med. Mycol. 49(Suppl. 1), S125–S136 10.3109/13693786.2010.516408 20840014

[B58] Leal S. M. Jr. Cowden S. Hsia Y. C. Ghannoum M. A. Momany M. Pearlman E. (2010). Distinct roles for Dectin-1 and TLR4 in the pathogenesis of *Aspergillus fumigatus* keratitis. PLoS Pathog. 6:e1000976 10.1371/journal.ppat.1000976 20617171 PMC2895653

[B59] Leibundgut-Landmann S. Wuthrich M. Hohl T. M. (2012). Immunity to fungi. Curr. Opin. Immunol. 24, 449–458 10.1016/j.coi.2012.04.007 22613091 PMC3538869

[B60] Levitz S. M. (2010). Innate recognition of fungal cell walls. PLoS Pathog. 6:e1000758 10.1371/journal.ppat.1000758 20421940 PMC2858700

[B61] Lewis L. E. Bain J. M. Lowes C. Gillespie C. Rudkin F. M. Gow N. A. (2012). Stage specific assessment of *Candida albicans* phagocytosis by macrophages identifies cell wall composition and morphogenesis as key determinants. PLoS Pathog. 8:e1002578 10.1371/journal.ppat.1002578 22438806 PMC3305454

[B62] Lilic D. (2012). Unravelling fungal immunity through primary immune deficiencies. Curr. Opin. Microbiol. 15, 420–426 10.1016/j.mib.2012.06.003 22818901

[B63] Lionakis M. S. Fischer B. G. Lim J. K. Swamydas M. Wan W. Richard Lee C. C. (2012). Chemokine receptor ccr1 drives neutrophil-mediated kidney immunopathology and mortality in invasive candidiasis. PLoS Pathog. 8:e1002865 10.1371/journal.ppat.1002865 22916017 PMC3420964

[B64] Lionakis M. S. Lim J. K. Lee C. C. Murphy P. M. (2010). Organ-specific innate immune responses in a mouse model of invasive candidiasis. J. Innate Immun. 3, 180–199 10.1159/000321157 21063074 PMC3072204

[B65] Loures F. V. Pina A. Felonato M. Calich V. L. (2009). TLR2 is a negative regulator of Th17 cells and tissue pathology in a pulmonary model of fungal infection. J. Immunol. 183, 1279–1290 10.4049/jimmunol.0801599 19553529

[B66] Majer O. Bourgeois C. Zwolanek F. Lassnig C. Kerjaschki D. Mack M. (2012). Type I interferons promote fatal immunopathology by regulating inflammatory monocytes and neutrophils during Candida infections. PLoS Pathog. 8:e1002811 10.1371/journal.ppat.1002811 22911155 PMC3406095

[B67] Marcil A. Gadoury C. Ash J. Zhang J. Nantel A. Whiteway M. (2008). Analysis of *PRA1* and its relationship to *Candida albicans*-macrophage interactions. Infect. Immun. 76, 4345–4358 10.1128/IAI.00588-07 18625733 PMC2519410

[B68] Massberg S. Von Andrian U. H. (2009). Novel trafficking routes for hematopoietic stem and progenitor cells. Ann. N.Y. Acad. Sci. 1176, 87–93 10.1111/j.1749-6632.2009.04609.x 19796236 PMC2847888

[B69] Medzhitov R. Schneider D. S. Soares M. P. (2012). Disease tolerance as a defense strategy. Science 335, 936–941 10.1126/science.1214935 22363001 PMC3564547

[B70] Meier A. Kirschning C. J. Nikolaus T. Wagner H. Heesemann J. Ebel F. (2003). *Toll*-like receptor (TLR) 2 and TLR4 are essential for Aspergillus-induced activation of murine macrophages. Cell. Microbiol. 5, 561–570 12864815 10.1046/j.1462-5822.2003.00301.x

[B71] Miyazato A. Nakamura K. Yamamoto N. Mora-Montes H. M. Tanaka M. Abe Y. (2009). *Toll*-like receptor 9-dependent activation of myeloid dendritic cells by deoxynucleic acids from *Candida albicans*. Infect. Immun. 77, 3056–3064 10.1128/IAI.00840-08 19433551 PMC2708591

[B72] Moreira A. P. Cavassani K. A. Ismailoglu U. B. Hullinger R. Dunleavy M. P. Knight D. A. (2011). The protective role of TLR6 in a mouse model of asthma is mediated by IL-23 and IL-17A. J. Clin. Invest. 121, 4420–4432 10.1172/JCI44999 22005301 PMC3204826

[B73] Moretti S. Bellocchio S. Bonifazi P. Bozza S. Zelante T. Bistoni F. (2008). The contribution of PARs to inflammation and immunity to fungi. Mucosal Immunol. 1, 156–168 10.1038/mi.2007.13 19079173

[B74] Murciano C. Moyes D. L. Runglall M. Islam A. Mille C. Fradin C. (2011). *Candida albicans* cell wall glycosylation may be indirectly required for activation of epithelial cell proinflammatory responses. Infect. Immun. 79, 4902–4911 10.1128/IAI.05591-11 21930756 PMC3232641

[B75] Naglik J. R. Moyes D. (2011). Epithelial cell innate response to *Candida albicans*. Adv. Dent. Res. 23, 50–55 10.1177/0022034511399285 21441481 PMC3144045

[B76] Nahum A. Dadi H. Bates A. Roifman C. M. (2011). The L412F variant of *Toll*-like receptor 3 (TLR3) is associated with cutaneous candidiasis, increased susceptibility to cytomegalovirus, and autoimmunity. J. Allergy Clin. Immunol. 127, 528–531 10.1016/j.jaci.2010.09.031 21093032

[B77] Nahum A. Dadi H. Bates A. Roifman C. M. (2012). The biological significance of TLR3 variant, L412F, in conferring susceptibility to cutaneous candidiasis, CMV and autoimmunity. Autoimmun. Rev. 11, 341–347 10.1016/j.autrev.2011.10.007 22024499

[B78] Nakamura K. Miyagi K. Koguchi Y. Kinjo Y. Uezu K. Kinjo T. (2006). Limited contribution of *Toll*-like receptor 2 and 4 to the host response to a fungal infectious pathogen, *Cryptococcus neoformans*. FEMS Immunol. Med. Microbiol. 47, 148–154 10.1111/j.1574-695X.2006.00078.x 16706798

[B79] Nakamura K. Miyazato A. Xiao G. Hatta M. Inden K. Aoyagi T. (2008). Deoxynucleic acids from *Cryptococcus neoformans* activate myeloid dendritic cells via a TLR9-dependent pathway. J. Immunol. 180, 4067–4074 18322216 10.4049/jimmunol.180.6.4067

[B80] Netea M. G. Gow N. A. Joosten L. A. Verschueren I. Van Der Meer J. W. Kullberg B. J. (2010). Variable recognition of *Candida albicans* strains by TLR4 and lectin recognition receptors. Med. Mycol. 48, 897–903 10.3109/13693781003621575 20166865

[B81] Netea M. G. Gow N. A. Munro C. A. Bates S. Collins C. Ferwerda G. (2006). Immune sensing of *Candida albicans* requires cooperative recognition of mannans and glucans by lectin and *Toll*-like receptors. J. Clin. Invest. 116, 1642–1650 10.1172/JCI27114 16710478 PMC1462942

[B82] Netea M. G. Sutmuller R. Hermann C. Van Der Graaf C. A. Van Der Meer J. W. Van Krieken J. H. (2004). *Toll*-like receptor 2 suppresses immunity against *Candida albicans* through induction of IL-10 and regulatory T cells. J. Immunol. 172, 3712–3718 15004175 10.4049/jimmunol.172.6.3712

[B83] Netea M. G. Quintin J. Van Der Meer J. W. (2011). Trained immunity: a memory for innate host defense. Cell Host Microbe 9, 355–361 10.1016/j.chom.2011.04.006 21575907

[B84] Netea M. G. Warris A. Van Der Meer J. W. Fenton M. J. Verver-Janssen T. J. Jacobs L. E. (2003). *Aspergillus fumigatus* evades immune recognition during germination through loss of Toll-like receptor-4-mediated signal transduction. J. Infect. Dis. 188, 320–326 10.1086/376456 12854089

[B85] Netea M. G. Wijmenga C. O'Neill L. A. (2012). Genetic variation in *Toll*-like receptors and disease susceptibility. Nat. Immunol. 13, 535–542 10.1038/ni.2284 22610250

[B86] Nickerson K. M. Christensen S. R. Shupe J. Kashgarian M. Kim D. Elkon K. (2010). TLR9 regulates TLR7- and MyD88-dependent autoantibody production and disease in a murine model of lupus. J. Immunol. 184, 1840–1848 10.4049/jimmunol.0902592 20089701 PMC4098568

[B87] Okagaki L. H. Strain A. K. Nielsen J. N. Charlier C. Baltes N. J. Chretien F. (2010). Cryptococcal cell morphology affects host cell interactions and pathogenicity. PLoS Pathog. 6:e1000953 10.1371/journal.ppat.1000953 20585559 PMC2887476

[B88] Ozinsky A. Underhill D. M. Fontenot J. D. Hajjar A. M. Smith K. D. Wilson C. B. (2000). The repertoire for pattern recognition of pathogens by the innate immune system is defined by cooperation between *toll*-like receptors. Proc. Natl. Acad. Sci. U.S.A. 97, 13766–13771 10.1073/pnas.250476497 11095740 PMC17650

[B89] Pandiyan P. Conti H. R. Zheng L. Peterson A. C. Mathern D. R. Hernandez-Santos N. (2011). CD4(+)CD25(+)Foxp3(+) regulatory T cells promote Th17 cells *in vitro* and enhance host resistance in mouse *Candida albicans* Th17 cell infection model. Immunity 34, 422–434 10.1016/j.immuni.2011.03.002 21435589 PMC3258585

[B90] Perlin D. S. (2011). Current perspectives on echinocandin class drugs. Future Microbiol. 6, 441–457 10.2217/fmb.11.19 21526945 PMC3913534

[B91] Pfaller M. A. (2012). Antifungal drug resistance: mechanisms, epidemiology, and consequences for treatment. Am. J. Med. 125, S3–S13 10.1016/j.amjmed.2011.11.001 22196207

[B92] Pfaller M. A. Diekema D. J. (2007). Epidemiology of invasive candidiasis: a persistent public health problem. Clin. Microbiol. Rev. 20, 133–163 10.1128/CMR.00029-06 17223626 PMC1797637

[B93] Pfaller M. A. Diekema D. J. (2010). Epidemiology of invasive mycoses in North America. Crit. Rev. Microbiol. 36, 1–53 10.3109/10408410903241444 20088682

[B94] Plantinga T. S. Johnson M. D. Scott W. K. Van De Vosse E. Velez Edwards D. R. Smith P. B. (2012). *Toll*-like receptor 1 polymorphisms increase susceptibility to candidemia. J. Infect. Dis. 205, 934–943 10.1093/infdis/jir867 22301633 PMC3282566

[B95] Puel A. Cypowyj S. Bustamante J. Wright J. F. Liu L. Lim H. K. (2011). Chronic mucocutaneous candidiasis in humans with inborn errors of interleukin-17 immunity. Science 332, 65–68 10.1126/science.1200439 21350122 PMC3070042

[B96] Qiu P. Pan P. C. Govind S. (1998). A role for the Drosophila Toll/Cactus pathway in larval hematopoiesis. Development 125, 1909–1920 9550723 10.1242/dev.125.10.1909

[B97] Quintin J. Saeed S. Martens J. H. Giamarellos-Bourboulis E. J. Ifrim D. C. Logie C. (2012). *Candida albicans* infection affords protection against reinfection via functional reprogramming of monocytes. Cell Host Microbe 12, 223–232 10.1016/j.chom.2012.06.006 22901542 PMC3864037

[B98] Ramaprakash H. Ito T. Standiford T. J. Kunkel S. L. Hogaboam C. M. (2009). *Toll*-like receptor 9 modulates immune responses to *Aspergillus fumigatus* conidia in immunodeficient and allergic mice. Infect. Immun. 77, 108–119 10.1128/IAI.00998-08 18936185 PMC2612288

[B99] Ramirez-Ortiz Z. G. Specht C. A. Wang J. P. Lee C. K. Bartholomeu D. C. Gazzinelli R. T. (2008). *Toll*-like receptor 9-dependent immune activation by unmethylated CpG motifs in *Aspergillus fumigatus* DNA. Infect. Immun. 76, 2123–2129 10.1128/IAI.00047-08 18332208 PMC2346696

[B100] Rehli M. (2002). Of mice and men: species variations of *Toll*-like receptor expression. Trends Immunol. 23, 375–378 12133792 10.1016/s1471-4906(02)02259-7

[B101] Rivera A. Ro G. Van Epps H. L. Simpson T. Leiner I. Sant'Angelo D. B. (2006). Innate immune activation and CD4+ T cell priming during respiratory fungal infection. Immunity 25, 665–675 10.1016/j.immuni.2006.08.016 17027299

[B102] Roeder A. Kirschning C. J. Schaller M. Weindl G. Wagner H. Korting H. C. (2004). Induction of nuclear factor-κ B and c-Jun/activator protein-1 via *Toll*-like receptor 2 in macrophages by antimycotic-treated *Candida albicans*. J. Infect. Dis. 190, 1318–1326 10.1086/423854 15346344

[B103] Romani L. (2011). Immunity to fungal infections. Nat. Rev. Immunol. 11, 275–288 10.1038/nri2939 21394104

[B104] Roy R. M. Klein B. S. (2012). Dendritic cells in antifungal immunity and vaccine design. Cell Host Microbe 11, 436–446 10.1016/j.chom.2012.04.005 22607797 PMC3401965

[B105] Rubino I. Coste A. Le Roy D. Roger T. Jaton K. Boeckh M. (2012). Species-specific recognition of *Aspergillus fumigatus* by *Toll*-like receptor 1 and *Toll*-like receptor 6. J. Infect. Dis. 205, 944–954 10.1093/infdis/jir882 22315281

[B106] Salvenmoser S. Seidler M. J. Dalpke A. Muller F. M. (2010). Effects of caspofungin, *Candida albicans* and *Aspergillus fumigatus* on *toll*-like receptor 9 of GM-CSF-stimulated PMNs. FEMS Immunol. Med. Microbiol. 60, 74–77 10.1111/j.1574-695X.2010.00720.x 20626764

[B107] Savage D. C. Dubos R. J. (1967). Localization of indigenous yeast in the murine stomach. J. Bacteriol. 94, 1811–1816 16562156 10.1128/jb.94.6.1811-1816.1967 PMC276909

[B108] Schneider D. S. Ayres J. S. (2008). Two ways to survive infection: what resistance and tolerance can teach us about treating infectious diseases. Nat. Rev. Immunol. 8, 889–895 10.1038/nri2432 18927577 PMC4368196

[B109] Seider K. Brunke S. Schild L. Jablonowski N. Wilson D. Majer O. (2011). The facultative intracellular pathogen *Candida glabrata* subverts macrophage cytokine production and phagolysosome maturation. J. Immunol. 187, 3072–3086 10.4049/jimmunol.1003730 21849684

[B110] Shoham S. Huang C. Chen J. M. Golenbock D. T. Levitz S. M. (2001). *Toll*-like receptor 4 mediates intracellular signaling without TNF-alpha release in response to *Cryptococcus neoformans* polysaccharide capsule. J. Immunol. 166, 4620–4626 11254720 10.4049/jimmunol.166.7.4620

[B111] Sica A. Mantovani A. (2012). Macrophage plasticity and polarization: *in vivo* veritas. J. Clin. Invest. 122, 787–795 10.1172/JCI59643 22378047 PMC3287223

[B112] Smeekens S. P. Van De Veerdonk F. L. Van Der Meer J. W. Kullberg B. J. Joosten L. A. Netea M. G. (2010). The Candida Th17 response is dependent on mannan- and beta-glucan-induced prostaglandin E2. Int. Immunol. 22, 889–895 10.1093/intimm/dxq442 21059767

[B113] Sorci G. Giovannini G. Riuzzi F. Bonifazi P. Zelante T. Zagarella S. (2011). The danger signal S100B integrates pathogen- and danger-sensing pathways to restrain inflammation. PLoS Pathog. 7:e1001315 10.1371/journal.ppat.1001315 21423669 PMC3053348

[B114] Sorgi C. A. Secatto A. Fontanari C. Turato W. M. Belanger C. De Medeiros A. I. (2009). *Histoplasma capsulatum* cell wall beta-glucan induces lipid body formation through CD18, TLR2, and dectin-1 receptors: correlation with leukotriene B4 generation and role in HIV-1 infection. J. Immunol. 182, 4025–4035 10.4049/jimmunol.0801795 19299700

[B115] Stuart L. M. Ezekowitz R. A. (2005). Phagocytosis: elegant complexity. Immunity 22, 539–550 10.1016/j.immuni.2005.05.002 15894272

[B116] Takahara K. Tokieda S. Nagaoka K. Inaba K. (2012). Efficient capture of *Candida albicans* and zymosan by SIGNR1 augments TLR2-dependent TNF-alpha production. Int. Immunol. 24, 89–96 10.1093/intimm/dxr103 22207132

[B117] Tanaka M. Ishii K. Nakamura Y. Miyazato A. Maki A. Abe Y. (2011). *Toll*-like receptor 9-dependent activation of bone marrow-derived dendritic cells by URA5 DNA from *Cryptococcus neoformans.* Infect. Immun. 80, 778–786 10.1128/IAI.05570-11 22104112 PMC3264295

[B118] Tessarolli V. Gasparoto T. H. Lima H. R. Figueira E. A. Garlet T. P. Torres S. A. (2010). Absence of TLR2 influences survival of neutrophils after infection with *Candida albicans*. Med. Mycol. 48, 129–140 10.3109/13693780902964339 19468929

[B119] Tierney L. Linde J. Muller S. Brunke S. Molina J. C. Hube B. (2012). An interspecies regulatory network inferred from simultaneous RNA-seq of *Candida albicans* invading innate immune cells. Front. Microbiol. 3:85 10.3389/fmicb.2012.00085 22416242 PMC3299011

[B120] Van Der Graaf C. Kullberg B. J. Joosten L. Verver-Jansen T. Jacobs L. Van Der Meer J. W. (2005). Functional consequences of the Asp299Gly *Toll*-like receptor-4 polymorphism. Cytokine 30, 264–268 10.1016/j.cyto.2005.02.001 15927851

[B121] Van Der Graaf C. A. Netea M. G. Morre S. A. Den Heijer M. Verweij P. E. Van Der Meer J. W. (2006). *Toll*-like receptor 4 Asp299Gly/Thr399Ile polymorphisms are a risk factor for Candida bloodstream infection. Eur. Cytokine Netw. 17, 29–34 16613760

[B122] Van De Veerdonk F. L. Kullberg B. J. Van Der Meer J. W. M. Gow N. A. R. Netea M. G. (2008a). Host-microbe interactions: innate pattern recognition of fungal pathogens. Curr. Opin. Microbiol. 11, 305–312 10.1016/j.mib.2008.06.002 18602019

[B123] Van De Veerdonk F. L. Netea M. G. Jansen T. J. Jacobs L. Verschueren I. Van Der Meer J. W. (2008b). Redundant role of TLR9 for anti-*Candida* host defense. Immunobiology 213, 613–620 10.1016/j.imbio.2008.05.002 18950591

[B124] Villamon E. Gozalbo D. Roig P. Murciano C. O'Connor J. E. Fradelizi D. (2004). Myeloid differentiation factor 88 (MyD88) is required for murine resistance to *Candida albicans* and is critically involved in *Candida*-induced production of cytokines. Eur. Cytokine Netw. 15, 263–271 15542452

[B125] Viriyakosol S. Fierer J. Brown G. D. Kirkland T. N. (2005). Innate immunity to the pathogenic fungus *Coccidioides posadasii* is dependent on *Toll*-like receptor 2 and Dectin-1. Infect. Immun. 73, 1553–1560 10.1128/IAI.73.3.1553-1560.2005 15731053 PMC1064940

[B126] Von Bernuth H. Picard C. Jin Z. Pankla R. Xiao H. Ku C. L. (2008). Pyogenic bacterial infections in humans with MyD88 deficiency. Science 321, 691–696 10.1126/science.1158298 18669862 PMC2688396

[B127] Wang J. Shao Y. Bennett T. A. Shankar R. A. Wightman P. D. Reddy L. G. (2006). The functional effects of physical interactions among *Toll*-like receptors 7, 8, and 9. J. Biol. Chem. 281, 37427–37434 10.1074/jbc.M605311200 17040905

[B128] Wang J. P. Lee C. K. Akalin A. Finberg R. W. Levitz S. M. (2011). Contributions of the MyD88-dependent receptors IL-18R, IL-1R, and TLR9 to host defenses following pulmonary challenge with *Cryptococcus neoformans*. PLoS ONE 6:e26232 10.1371/journal.pone.0026232 22039448 PMC3198470

[B129] Wang S. H. Zhang C. Lasbury M. E. Liao C. P. Durant P. J. Tschang D. (2008). Decreased inflammatory response in *Toll*-like receptor 2 knockout mice is associated with exacerbated *Pneumocystis pneumonia*. Microbes Infect. 10, 334–341 10.1016/j.micinf.2007.12.014 18400546 PMC2423425

[B130] Weindl G. Naglik J. R. Kaesler S. Biedermann T. Hube B. Korting H. C. (2007). Human epithelial cells establish direct antifungal defense through TLR4-mediated signaling. J. Clin. Invest. 117, 3664–3672 10.1172/JCI28115 17992260 PMC2066194

[B131] Weindl G. Wagener J. Schaller M. (2011). Interaction of the mucosal barrier with accessory immune cells during fungal infection. Int. J. Med. Microbiol. 301, 431–435 10.1016/j.ijmm.2011.04.011 21550846

[B132] Wuthrich M. Deepe G. S. Jr. Klein B. (2012a). Adaptive immunity to fungi. Annu. Rev. Immunol. 30, 115–148 10.1146/annurev-immunol-020711-074958 22224780 PMC3584681

[B133] Wuthrich M. Ersland K. Sullivan T. Galles K. Klein B. S. (2012b). Fungi subvert vaccine T cell priming at the respiratory mucosa by preventing chemokine-induced influx of inflammatory monocytes. Immunity 36, 680–692 10.1016/j.immuni.2012.02.015 22483803 PMC3334432

[B134] Wuthrich M. Gern B. Hung C. Y. Ersland K. Rocco N. Pick-Jacobs J. (2011). Vaccine-induced protection against 3 systemic mycoses endemic to North America requires Th17 cells in mice. J. Clin. Invest. 121, 554–568 10.1172/JCI43984 21206087 PMC3026727

[B135] Yamamoto H. Abe Y. Miyazato A. Tanno D. Tanaka M. Miyasaka T. (2011). *Cryptococcus neoformans* suppresses the activation of bone marrow-derived dendritic cells stimulated with its own DNA, but not with DNA from other fungi. FEMS Immunol. Med. Microbiol. 63, 363–372 10.1111/j.1574-695X.2011.00859.x 22092563

[B136] Yanez A. Flores A. Murciano C. O'Connor J. E. Gozalbo D. Gil M. L. (2010). Signalling through TLR2/MyD88 induces differentiation of murine bone marrow stem and progenitor cells to functional phagocytes in response to *Candida albicans*. Cell. Microbiol. 12, 114–128 10.1111/j.1462-5822.2009.01382.x 19747212

[B137] Yanez A. Megias J. O'Connor J. E. Gozalbo D. Gil M. L. (2011). *Candida albicans* induces selective development of macrophages and monocyte derived dendritic cells by a TLR2 dependent signalling. PLoS ONE 6:e24761 10.1371/journal.pone.0024761 21935459 PMC3174213

[B138] Yanez A. Murciano C. O'Connor J. E. Gozalbo D. Gil M. L. (2009). *Candida albicans* triggers proliferation and differentiation of hematopoietic stem and progenitor cells by a MyD88-dependent signaling. Microbes Infect. 11, 531–535 10.1016/j.micinf.2009.01.011 19217944

[B139] Yauch L. E. Mansour M. K. Shoham S. Rottman J. B. Levitz S. M. (2004). Involvement of CD14, *toll*-like receptors 2 and 4, and MyD88 in the host response to the fungal pathogen *Cryptococcus neoformans in vivo*. Infect. Immun. 72, 5373–5382 10.1128/IAI.72.9.5373-5382.2004 15322035 PMC517466

[B140] Zaragoza O. Garcia-Rodas R. Nosanchuk J. D. Cuenca-Estrella M. Rodriguez-Tudela J. L. Casadevall A. (2010). Fungal cell gigantism during mammalian infection. PLoS Pathog. 6:e1000945 10.1371/journal.ppat.1000945 20585557 PMC2887474

[B141] Zelante T. De Luca A. D'Angelo C. Moretti S. Romani L. (2009). IL-17/Th17 in anti-fungal immunity: what's new? Eur. J. Immunol. 39, 645–648 10.1002/eji.200839102 19283705

[B142] Zhang Y. Wang F. Bhan U. Huffnagle G. B. Toews G. B. Standiford T. J. (2010). TLR9 signaling is required for generation of the adaptive immune protection in *Cryptococcus neoformans*-infected lungs. Am. J. Pathol. 177, 754–765 10.2353/ajpath.2010.091104 20581055 PMC2913381

